# The Incidence and Clinical Characteristics of Infective Endocarditis in Children: A Five-Year, Single-Centre Retrospective Evaluation

**DOI:** 10.7759/cureus.31747

**Published:** 2022-11-21

**Authors:** Münevver Yılmaz, Dolunay Gürses, Özge Kahraman

**Affiliations:** 1 Pediatric Cardiology, Faculty of Medicine, Pamukkale University, Denizli, TUR; 2 Pediatrics, Faculty of Medicine, Pamukkale University, Denizli, TUR

**Keywords:** incidence, congenital heart disease (chd), blood culture, child, infective endocarditis

## Abstract

Introduction: Infective endocarditis (IE) is a rare disease with high mortality and morbidity. In recent years, an increase in the frequency of infective endocarditis has been observed due to the increase in the survival of cases with congenital heart diseases (CHDs) and the use of central catheters. In addition to revealing the incidence of IE in our clinic, this study aimed to evaluate the demographic characteristics, predisposing factors, clinical, laboratory, microbiological, and echocardiographic findings, and the complications of follow-up of our patients diagnosed with IE in light of the literature.

Material and methods: Thirteen patients with IE who were hospitalized in Pediatric Cardiology Clinic, Pamukkale University Medical Faculty Hospital between January 2016 and August 2021 were retrospectively reviewed. The patients included in the study were evaluated in terms of demographic characteristics, predisposing factors, clinical, laboratory and microbiological findings, echocardiography data, surgical intervention needs, and complications. The incidence of IE in our clinic was defined as the rate of IE among patients admitted to the hospital and reported as the number of patients with IE per 100,000 hospital admissions.

Results: The median age of these 13 patients was 11 (7-14) years, and the male/female ratio was 6/7. The five-year IE incidence in Pediatric Cardiology Clinic, Pamukkale University Medical Faculty Hospital, was found to be 2.5 in approximately 100,000 hospital admissions. A predisposing factor was detected in all patients. Six patients (46%) had CHDs, and four patients (31%) had acquired heart disease. The other three (23%) patients were receiving immunosuppressive therapy, and these patients had long-term port catheters. Eight (62%) patients had positive blood cultures. *Streptococcus viridans, Streptococcus gordonii, Staphylococcus aureus, *coagulase-negative*Staphylococcus,* *Brucella spp,* *and* *Candida albicans* were isolated in the blood cultures of these patients. IE-related complications developed in seven (54%) patients. Five (38%) had heart failure, three patients (23%) had thromboembolic events, two (15%) had glomerulonephritis, and one (8%) had thrombophlebitis. Four patients were referred to early surgery. Two patients with recurrent IE attacks were referred to surgery after their treatment was completed.

Conclusion: The incidence of IE has shown an increase recently with increased rates of survival attributable to corrective surgeries performed for congenital heart diseases, increased prosthetic materials used in cardiac surgeries, and increased use of permanent catheters. In our study, the incidence of IE was found to be 2.5 in 100,000 hospital admissions. Our results have shown that rheumatic heart diseases, besides CHDs, are still an important risk factor for Turkey. Due to the low number of cases in IE studies in the pediatric population, there is a need for further studies to be conducted in large series in this field.

## Introduction

Infective endocarditis (IE) is a rare disease with high mortality and morbidity [1-4]. In recent years, an increase in the frequency of IE and changes in predisposing factors have been observed due to the increase in the survival and central catheter use of patients with congenital heart diseases (CHDs) [1-6]. While 30-50% of IE cases were seen due to previous rheumatic heart disease in the 1970s; CHDs have become the most common predisposing factor due to the increase in the survival of CHDs in recent years [1,7,8]. However, IE is also seen in children without heart disease. It has been reported that these children often have a central catheter and/or receive immunosuppressive treatment [1,2].

Despite the advances in diagnostic methods, effective antimicrobial treatments, and advances in surgical techniques, the morbidity and mortality of IE are still high. Due to the difficulty of conducting a randomized controlled trial for IE patients, there are still doubts about the diagnosis and treatment [1,2,9,10]. In addition to revealing the incidence of IE in our clinic, this study aimed to evaluate the demographic characteristics, predisposing factors, clinical, laboratory, microbiological and echocardiographic findings, and the complications of follow-up of our patients diagnosed with IE with the literature.

## Materials and methods

Thirteen patients with IE who were hospitalized in Pediatric Cardiology Clinic of Pamukkale University Medical Faculty Hospital between January 2016 and August 2021 were retrospectively examined. The patients included in the study were evaluated in terms of demographic characteristics, predisposing factors, clinical, laboratory and microbiological findings, echocardiography data, surgical intervention needs and complications. Transthoracic echocardiographic evaluation was performed with a GE VingmedVivid Pro 7 (GE Vingmed Ultrasound Horten, Norway) echocardiography device with 2.5-3.5 MHz and 2.7-8 MHz probes. The diagnosis of IE was made according to the Modified Duke Criteria [1,2]. Recurrent IE attacks were defined as attacks that occurred more than six months after the first attack or a different pathogen was detected [8]. To calculate the incidence of IE, the number of patients who applied to Pamukkale University Medical Faculty Hospital Pediatric Clinics between January 2016 and August 2021 was determined from electronic records. Before the study, approval was obtained from the Pamukkale University Faculty of Medicine Non-Interventional Clinical Research Ethics Committee.

In the statistical evaluation of the data, the Statistical Package Social Sciences for Windows SPSS, version 18.0 (SPSS Inc., Chicago, IL, USA) software and descriptive statistics were used. The results were obtained as numbers and percentages for categorical data and as median (interquartile range) for continuous data. The incidence of IE in our clinic was defined as the rate of IE among patients admitted to the hospital and reported as the number of patients with IE per 100,000 hospital admissions [11].

## Results

**Table 1:** Demographic, clinical and echocardiographic data of our patients. M: Male, F: Female, FT: Fallot Tetralogy, VSD: Ventricular Septal Defect, AVSD: Atrioventricular septal defect, AS: Aortic stenosis

	Sex	Age (years)	Duke Criteria	Predisposing Factor	Vegetation Localization	Fever	Microbiological Evidence	Early Surgical Intervention	Complications
Case 1	M	17	1 major, 4 minor	Pumoner Atresia FT	Conduit with pulmonary valve and mitral valve	Yes	No	No	Glomerulonephritis heart failure
Case 2	F	13	1 major, 2 minor	Bicuspid aortic valve	Aortic valve	Yes	Coagulase negative staphylococcus	Yes	Septic embolism heart failure
Case 3	F	8	1 major, 3 minor	VSD	Right ventricle	Yes	S. Gordonii	Yes	No
Case 4	M	7	1 major, 2 minor	Rheumatic heart disease	Mitral valve	Yes	No	No	No
Case 5	F	17	1 major, 1 minor	Dilated cardiomyopathy	Mitral valve	No	No	No	Septic embolism heart failure
Case 6	F	5	1 major, 1 minor	Operated AVSD	Mitral valve	No	No	No	Heart failure
Case 7	M	7	2 major, 2 minor	Bicuspid aortic valve AS+Aortic valve replacement	Prosthetic aortic valve	Yes	S. Viridans	No	Thrombophlebitis
Case 8	F	12	2 major, 3 minor	Operated truncus arteriosus type 1	Conduit with pulmonary valve and aortic valve	Yes	S. Aureus	No	Glomerulonephritis heart failure
Case 9	F	12	1 major, 2 minor	Rheumatic heart disease	Mitral and aortic valve	Yes	Brucella	Yes	No
Case 10	M	10	2 major, 2 minor	Rheumatic heart disease	Mitral valve	Yes	S. Viridans	No	No
Case 11	M	3	2 major, 3 minor	Catheter, immunosuppression	Tricuspid valve	Yes	Candida	Yes	Pulmonary embolism
Case 12	F	3	1 major, 2 minor	Catheter, immunosuppression	Right atrium	Yes	No	No	No
Case 13	M	9	1 major, 2 minor	Catheter, immunosuppression	Right atrium	Yes	Coagulase negative staphylococcus	No	No

A total of 13 patients were diagnosed with IE between January 2016 and August 2021, and a total of 512,715 patients were admitted to pediatric clinics. The median age of the 13 patients was 11 (7-14) years, and the male/female ratio was 6/7. According to the Modified Duke Criteria, six (46%) patients were defined as having possible IE, and seven (54%) patients were defined as having definite IE. Two (15%) cases had recurrent IE attacks. The five-year IE incidence of Pamukkale University Medical Faculty Hospital Pediatric Clinics was found to be 2.5 in approximately 100,000 hospital admissions.

A predisposing factor was detected in all patients. Six patients (46%) had CHD, and four patients (31%) had acquired heart disease. The other three (23%) patients were receiving immunosuppressive therapy, and these patients had long-term port catheters. Two of these patients (15%) were followed up due to pre-B-cell acute lymphoblastic leukemia, and one (8%) was followed up due to rhabdomyosarcoma.

Four of six (31%) patients with CHDs had previously been operated on for heart disease, and three patients additionally had residual defects. Case 1 was operated on with the diagnosis of tetralogy of Fallot with pulmonary atresia. Moderate stenosis was detected in the conduit with the pulmonary valve in the outpatient clinic follow-ups. This patient had been treated for two attacks of IE with an interval of approximately one year. Case 6 had been operated on for an atrioventricular septal defect and had mitral valve insufficiency. Case 8 was operated on for truncus arteriosus type 1. In the outpatient follow-up, moderate aortic valve regurgitation and severe stenosis in the pulmonary valve conduit were observed. This patient, on the other hand, had been treated for two attacks of IE with an interval of approximately nine months. Case 7 had a bicuspid aortic valve, and had aortic valve replacement due to severe aortic stenosis. The other two patients were followed with the diagnosis of ventricular septal defect and bicuspid aortic valve (Case 2, Case 3). Three (23%) of the four patients with acquired heart disease were followed with the diagnosis of rheumatic heart disease and the other with the diagnosis of dilated cardiomyopathy (Table 1).

Eleven patients (85%) had a fever >38 °C at the time of admission. Patients were followed up with a temperature of >38 °C for a median of 10 (4-15) days. Two patients (15%) who did not have a fever in clinical follow-up had received antimicrobial treatment before admission. Pathological murmur was present at the time of admission in 10 (77%) patients who were followed up due to acquired and congenital heart disease before admission. An increase in the severity of the murmur was detected in two of these patients, who also had a murmur during their outpatient follow-up physical examinations. The patient followed up for rhabdomyosarcoma had a newly developed pansystolic murmur due to tricuspid valve insufficiency (Case 11). The SpO2 value at the time of admission was 68% in the patient who was being followed ups with operated pulmonary atresia Fallot tetralogy and had clubbing fingers (Case 1).

Acute phase reactants were high in all patients at the time of diagnosis. The median C-reactive protein level was 91 (38-305) mg/L, the median white blood cell count was 14.130 (10.200-19.970) K/µL, and the erythrocyte sedimentation rate was 71 (28-115) mm/h.

Eight (62%) patients had positive blood cultures. Blood culture was positive in four of six patients with CHDs as a predisposing factor. *Streptococcus viridans*, *Streptococcus gordonii*, *Staphylococcus aureus*, and coagulase-negative *Staphylococcus* were isolated in the blood cultures of these four patients. *Streptococcus viridans* and *Brucella* species were isolated in the blood cultures of two patients with acquired heart disease. Two of the three patients who received immunosuppressive therapy and had a port catheter had positive blood cultures. *Candida albicans* and coagulase-negative *Staphylococcus* were isolated in the blood cultures of these patients. Five (38%) patients who had negative blood cultures had received antimicrobial treatment before admission. Right-sided IE was detected in four patients (31%). Three of these patients (75%) had a catheter. Nine (69%) patients were observed to have left-sided IE (Table 1).

IE-related complications developed in seven (54%) patients. The most common complication was heart failure; five (38%) of our patients received parenteral inotropic treatment for heart failure. Three patients (23%) had thromboembolic events, two (15%) had glomerulonephritis, and one (8%) had thrombophlebitis (Table 1). In two of the patients who developed thromboembolic events, septic embolism was detected in the cranial imaging performed upon the observation of behavioral changes during follow-up. Case 11 was receiving chemotherapy for rhabdomyosarcoma and had a port catheter. He had vegetation on the tricuspid valve. A pulmonary embolism was detected in this patient who underwent thoracic computed tomography angiography due to tachypnea. In our patient, who had macroscopic hematuria at first attack admission, glomerulonephritis was detected, and immunosuppressive treatment was administered in addition to antimicrobial treatment (Case 8, Table 1).

All patients were administered appropriate antimicrobial treatment. In addition, six (46%) patients required surgical intervention due to IE. Three patients (23%) were referred to early surgery due to persistent vegetation despite appropriate antimicrobial treatment and one patient was referred due to pulmonary embolism and fungal endocarditis (Table 1). Two (15%) patients with recurrent IE attacks were referred to surgery after their treatment was completed (Case 1, Case 8). The median length of hospital stay of 13 patients was 46.5 (23-62) days. None of our patients died due to IE during the follow-up period. The demographic and echocardiographic findings of the patients are given in Table 1.

## Discussion

The incidence of IE is estimated to be 0.34-0.84 cases in every 100,000 children [1-4]. This rate has been reported as 2.2 cases in 10,000 children with CHDs [12]. The incidence of IE among children admitted to the hospital has been reported to be 5-12 cases in 100,000 admissions [13]. In a study, the incidence of IE in children admitted to the hospital in Turkey was found to be 10.3 cases in 100,000 admissions [14]. In our study, we calculated this rate as 2.5 cases in approximately 100,000 admissions.

Congenital and rheumatic heart diseases are the most common predisposing factors in IE. While rheumatic heart diseases were the most common risk factors in the early twentieth century, there has been a shift toward CHDs in etiology with the increase in survival in CHDs today [1,6,9,10]. In childhood, approximately 50-70% of patients with IE have reported the presence of CHDs as the underlying risk factor [7,12]. There is a study that found that children with CHDs have an average risk of 15-140 times higher risk of developing IE compared to the general population [12]. The studies conducted in Turkey have reported that the most common risk factor is CHDs, and 43-91% of children with IE have underlying CHDs. Various rates between 0% and 43% have been reported for rheumatic heart disease [14-18]. Our results support that CHDs are the first predisposing factor; however, rheumatic heart disease is still an important risk factor in our country.

Surgical interventions and prosthetic materials used in congenital and acquired heart diseases increase the risk of IE [1-5,7,19]. Although it has been reported that the risk of IE increases, especially in patients with prosthetic valves, more than 25% of patients have a history of valve surgery in natural valve IE [6]. In addition, children with repaired complex congenital heart disease have the highest risk of IE compared to other cardiac defects [5,7,8]. In our study, 31% of our patients had a history of cardiac surgery, and three of these patients had prosthetic valves or conduits.

In the literature, 14-26% of IEs develop structurally in normal hearts [1-6]. The majority of these children have a history of an underlying serious chronic disease, immunodeficiency, central catheter, or frequent invasive intervention (chemotherapy, nutrition, or hemodialysis). Children with cancer may have multiple risk factors, such as tumor-related immunosuppression, nutritional deficiency, chemotherapy, and the presence of central catheters. The frequency of IE has increased significantly in patients in this group in recent years [2-8,19]. Twenty-three percent of our IE cases did not have structural heart disease and all of these patients had a history of central catheterization and received chemotherapy due to cancer. Therefore, it should be kept in mind that IE may be seen in children with chronic diseases, especially those under cancer treatment, even if there is no underlying heart disease.

Although IE is most commonly of bacterial origin, fungi, viruses, and other microbiological agents can also cause endocarditis. Although blood cultures have a very important place in both the diagnosis and treatment of IE, 5-68% of patients had negative blood cultures. It has been reported that the reasons for this are the use of antibiotics before culture taking, infections caused by slow-growing microorganisms, or the use of inadequate microbiological techniques [7,20-23]. In our study, 38% of patients had negative blood cultures. All of these patients had a history of pre-culture antibiotic use.

Staphylococcal species and streptococcal species in children are the most common bacteria causing endocarditis [1-5,6,20]. In our study, 75% of the microorganisms, were isolated in the blood culture had been *Staphylococcus* species and *Streptococcus* species. Heart involvement has been reported to be as low as 1.5% in *Brucella* cases. Heart involvement, which is a rare complication in *Brucella* cases, has been reported to occur frequently in patients diagnosed with rheumatic heart disease [24]. In our study, *Brucella* species was isolated in the blood culture of a patient with rheumatic heart disease supports this finding.

Fungal endocarditis is a rare disease that occurs in patients with predisposing factors. It mainly occurs in immunocompromised patients who have received broad-spectrum antibiotic therapy and have heart valve replacement and/or pacemakers [19-21]. In our study, fungal IE was detected in a patient with a catheter followed up for rhabdomyosarcoma and receiving active chemotherapy. Empirical antimicrobial treatment should be started as soon as possible after the diagnosis of IE [1-5,22]. Surgical treatment should be planned in cases where antimicrobial treatment alone is considered insufficient. Surgical treatment is applied to 25-50% of IE patients [2,6,20,25]. Surgical treatment was required in 46% of our patients. Three patients (23%) were referred to early surgery due to persistent vegetation despite appropriate antimicrobial treatment, and one patient was referred to early surgery due to pulmonary embolism and fungal endocarditis.

Heart failure is the most common complication of IE and is one of the most common causes of surgical intervention requirements. The rate of heart failure is 42-66% in the literature. The main cause is insufficiency due to mitral valve and aortic valve involvement, and higher rates of heart failure have been detected in aortic valve involvement [1,7,22]. Heart failure developed in 38% of our patients; two of these patients had aortic valve involvement and three had mitral valve involvement.

Extracardiac complications are caused by embolization of cardiac vegetation, sepsis, and immune-mediated mechanisms (such as glomerulonephritis and vasculitis). Septic embolism is a life-threatening complication seen in 20-50% of patients. Embolic events may include the brain, lungs, peripheral vascular system, kidneys, and many other organs. IE, which includes the anterior leaflet of the mitral valve as well as staphylococcal or fungal infections, has a higher risk of embolization [1,13,22]. Pulmonary embolism is a serious complication of right-sided IE [1]. Two of our patients (15%) developed cerebral embolism and one developed pulmonary embolism. These patients had mitral valve endocarditis, *Staphylococcus*, and fungal infection, which are risk factors for embolism. Renal dysfunction can be seen in 6-30% of IE patients. This condition develops from vasculitis or glomerulonephritis due to immune complexes. In addition to antibiotic therapy for IE, glomerulonephritis may require the administration of immunosuppressive drugs [2,13]. In our case, a patient who developed glomerulonephritis required additional immunosuppressive therapy.

Recurrent IE attacks occur in approximately 2.5-9% of patients. The continuation of predisposing factors such as poor oral hygiene, prosthetic valve IE, chronic disease, central catheter, or in-heart devices significantly increases the likelihood of recurrent IE attacks [7,8]. IE recurred in two of our patients with pulmonary conduits and the patients were referred to surgery after treatment.

The limitations of the study are its retrospective nature and the small number of cases. Also, imaging modalities other than transthoracic echocardiography are not discussed. Further, the study was conducted in a single center of a country. Therefore, generalization of the results might not be feasible. 

## Conclusions

The incidence of IE has shown an increase recently with increased rates of survival attributable to corrective surgeries performed for congenital heart diseases, increased prosthetic materials used in cardiac surgeries and increased use of permanent catheters. In our study, the incidence of IE was found to be 2.5 in 100,000 hospital admissions. Our results have shown that rheumatic heart diseases, besides CHDs, are still an important risk factor for Turkey. Due to the low number of cases in IE studies in the pediatric population, there is a need for further studies to be conducted in large series in this field.

## References

[1] Cox DA, Tani LY (2020). Pediatric infective endocarditis: a clinical update. Pediatr Clin North Am.

[2] Eleyan L, Khan AA, Musollari G (2021). Infective endocarditis in paediatric population. Eur J Pediatr.

[3] Mahony M, Lean D, Pham L (2021). Infective endocarditis in children in Queensland, Australia: epidemiology, clinical features and outcome. Pediatr Infect Dis J.

[4] Jortveit J, Eskedal L, Klcovansky J, Døhlen G, Holmstrøm H (2019). Prevalence of infective endocarditis in children. Tidsskr Nor Laegeforen.

[5] Martin J, Lindgren C (2018). Infectious endocarditis prophylaxis in children. Pediatr Emerg Care.

[6] Khoo B, Buratto E, Fricke TA (2019). Outcomes of surgery for infective endocarditis in children: a 30-year experience. J Thorac Cardiovasc Surg.

[7] Vicent L, Luna R, Martínez-Sellés M (2022). Pediatric infective endocarditis: a literature review. J Clin Anal Med.

[8] Song SH, Bang JS, Han MS (2021). Changes in the etiology and clinical characteristics of pediatric infective endocarditis in South Korea. Pediatr Infect Dis J.

[9] Tseng WC, Chiu SN, Shao PL (2014). Changing spectrum of infective endocarditis in children: a 30 years experiences from a tertiary care center in Taiwan. Pediatr Infect Dis J.

[10] Day MD, Gauvreau K, Shulman S, Newburger JW (2009). Characteristics of children hospitalized with infective endocarditis. Circulation.

[11] Angsutararux T, Angkasekwinai N (2019). Cumulative incidence and mortality of infective endocarditis in Siriraj hospital-Thailand: a 10-year retrospective study. BMC Infect Dis.

[12] Jortveit J, Klcovansky J, Eskedal L, Birkeland S, Døhlen G, Holmstrøm H (2018). Endocarditis in children and adolescents with congenital heart defects: a Norwegian nationwide register-based cohort study. Arch Dis Child.

[13] Singh Y, Ganjoo N (2017). Infective endocarditis in children. Paediatr Child Health.

[14] Oncul M, Karakurt C, Elkiran O (2021). An evaluation of risk factors, clinical features, and follow-up findings of patients with infective endocarditis. Ann Med Res.

[15] Tavli V, Cevik BS, Saritas T, Mese T (2009). Retrospective evaluation of clinical, laboratory, and echocardiographic parameters of infective endocarditis in children (Article in Turkish). Gazi Med J.

[16] Kosger P, Keskin T, Kiztanir H, Ucar B (2020). Clinical characteristics, treatment approaches, and complications in children with infective endocarditis: a single center experience from Turkey. Osmangazi J Med.

[17] Irdem A, Başpınar O, Kervancıoğlu M, Şahin DA, Kılınç M (2012). A retrospective evaluation of patients with infective endocarditis (Article in Turkish). J Pediatr Inf.

[18] Yakut K, Ecevit Z, Tokel NK, Varan B, Ozkan M (2021). Infective endocarditis in childhood: a single-center experience of 18 years. Braz J Cardiovasc Surg.

[19] Bezerra LS, Silva JA, Santos-Veloso MA, Lima SG, Chaves-Markman ÂV, Jucá MB (2020). Antifungal efficacy of amphotericin B in Candida albicans endocarditis therapy: systematic review. Braz J Cardiovasc Surg.

[20] Luca AC, Curpan AS, Adumitrachioaiei H (2021). Difficulties in diagnosis and therapy of infective endocarditis in children and adolescents-cohort study. Healthcare (Basel).

[21] Xu H, Cai S, Dai H (2016). Characteristics of infective endocarditis in a tertiary hospital in East China. PLoS One.

[22] Baltimore RS, Gewitz M, Baddour LM (2015). Infective endocarditis in childhood: 2015 update: a scientific statement from the American Heart Association. Circulation.

[23] Liesman RM, Pritt BS, Maleszewski JJ, Patel R (2017). Laboratory diagnosis of infective endocarditis. J Clin Microbiol.

[24] Koruk ST, Erdem H, Koruk I (2012). Management of Brucella endocarditis: results of the Gulhane study. Int J Antimicrob Agents.

[25] Carrillo SA, Duenas H, Blaney C, Eisner M, Nandi D, McConnell PI (2022). Surgical outcomes of infective endocarditis in pediatrics: Moving the needle to a contemporary, multidisciplinary approach. J Thorac Cardiovasc Surg.

